# A new regulator of caveolae signalling

**DOI:** 10.7554/eLife.01428

**Published:** 2013-09-24

**Authors:** Alan J Whitmarsh

**Affiliations:** 1 **Alan J Whitmarsh** is at the Faculty of Life Sciences, University of Manchester, Manchester, United Kingdomalan.j.whitmarsh@manchester.ac.uk

**Keywords:** Cavin-3, hSRBC, PRKCDBP, ERK, Akt, aerobic glycolysis, Human, Mouse

## Abstract

Cavin-3 regulates metabolism and cell proliferation by coordinating the activities of growth factor signalling cascades.

**Related research article** Hernandez VJ, Weng J, Ly P, Pompey S, Dong H, Mishra L, Schwarz M, Anderson RGW, Michaely P. 2013. Cavin-3 dictates the balance between ERK and Akt signaling. *eLife* **2**:e00905. doi: 10.7554/eLife.00905

**Image** The plasma membranes of most cells contain pits called caveolae (arrows)

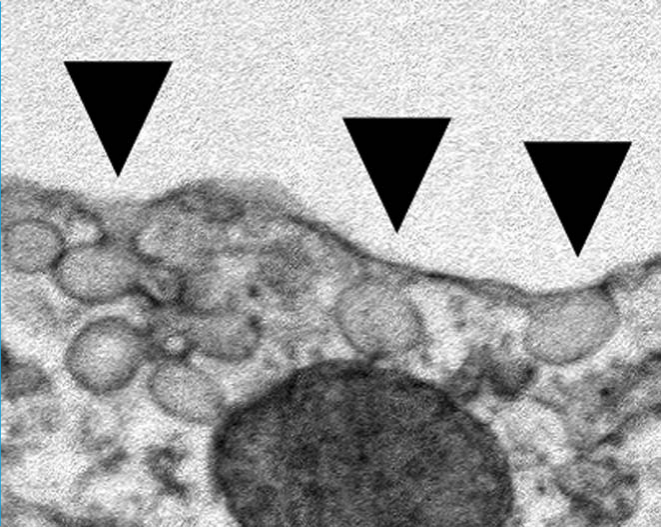

More than sixty years ago, electron microscopy revealed that the plasma membrane of the cell contains specialized domains with distinct morphologies. These include small invaginations called caveolae that are found in the membranes of most cell types. For many years the functions of caveolae (‘little caves’ in Latin) remained elusive, but recent studies have implicated them in a range of processes, including lipid homeostasis, endocytosis and the protection of cells from mechanical stress (Parton and del Pozo, 2013). Now, in *eLife*, Peter Michaely at the University of Texas Southwestern Medical Centre and colleagues report that a protein called cavin-3, which is localized to caveolae, is involved in regulating the activities of two major intracellular signalling pathways and in controlling metabolism and cell proliferation (Hernandez et al., 2013).

Caveolae are formed through the actions of caveolins, a family of proteins that aggregate at the interface between the cytoplasm and the plasma membrane (Parton and del Pozo, 2013). Caveolins form complexes with additional proteins including members of the cavin family. Of these, cavin-1 is the best characterized and is known to be required for caveolae formation (Hansen and Nichols, 2010). Cavin-3 was originally identified as a binding partner and substrate of the enzyme PKC (protein kinase C), and was later shown to be associated with caveolae (Izumi et al., 1997; McMahon et al., 2009).

To address the role of cavin-3 in caveolae function and to determine its biological significance, Michaely and colleagues—including Victor Hernandez as first author—used biochemical and loss of function approaches in cultured fibroblasts, and also generated mice that lack cavin-3. They found that cavin-3 links caveolae with the actin cytoskeleton—the network of scaffolding that maintains a cell’s structure and shape. They provide evidence that this may be important for maintaining caveolae at the plasma membrane, and also for facilitating growth factor signalling through a cascade known as the ERK MAP kinase pathway.

Hernandez et al. showed that loss of cavin-3 expression triggers a reduction in ERK signalling, and that this change coincides with an increase in signalling by another protein kinase called Akt, which is a key regulator of cell growth and survival. The reciprocal changes in the activities of these two pathways may be linked. Thus, down-regulation of ERK activity leads to reduced expression of the transcription factor EGR1 (Early Growth Response protein 1) and, subsequently, to reduced expression of the gene that codes for a protein called PTEN. Since PTEN inhibits the action of Akt, the down-regulation of ERK ultimately leads to the up-regulation of Akt (Figure 1). Additional mechanisms for up-regulating Akt activity that are dependent on the ERK-EGR1 pathway clearly exist but have yet to be identified.

**Figure 1.**
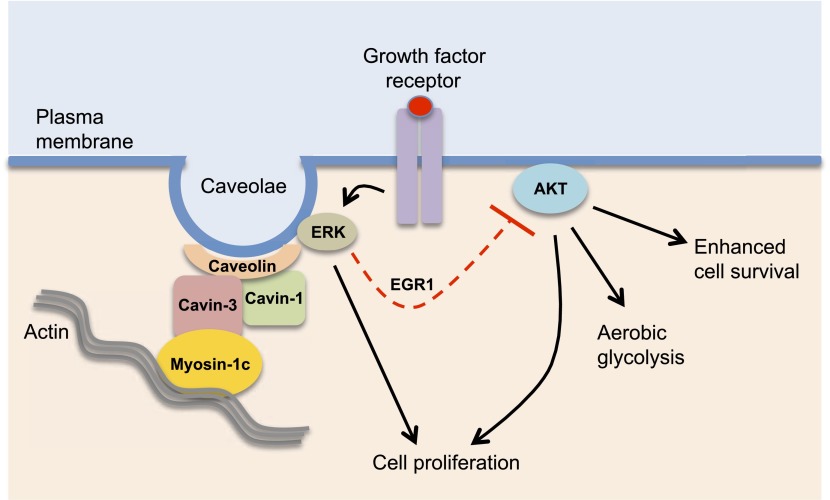
Cavin-3 regulates caveolae signalling. Cavin-3 forms a complex with cavin-1 and caveolin and helps stabilize caveolae at the plasma membrane by linking them to the actin cytoskeleton via the protein myosin-1c. Caveolae at the plasma membrane can promote cellular growth by recruiting components of the ERK MAP kinase pathway and facilitating its activation by growth factors. ERK signalling can suppress (dashed red line) the activity of Akt, a protein kinase that is involved in many processes inside the cell. The loss of cavin-3 leads to enhanced Akt activity, which promotes cell survival and aerobic glycolysis, which may help explain why cavin-3 has tumour suppressor activity.

How does this signalling switch alter cellular function? It renders cells less susceptible to programmed cell death (apoptosis) and enhances aerobic glycolysis and cell proliferation. Aerobic glycolysis is the conversion of glucose to lactate despite oxygen being available—a phenomenon known as the ‘Warburg effect’—and is a hallmark of some rapidly dividing cancer cells (Cairns et al., 2011). Loss of cavin-3 protein has been reported in epithelial and glial-derived cancers, indicating that cavin-3 may be a tumour suppressor (Xu et al., 2001). The work of Hernandez et al. suggests that one way in which cavin-3 could suppress tumour formation is by limiting aerobic glycolysis. They demonstrate that introducing cavin-3 into a lung carcinoma cell line in which cavin-3 is absent, leads to enhanced ERK activation and a reduction in both Akt signalling and aerobic glycolysis. While cavin-3 knock-out mice do not generate tumours spontaneously, the loss of cavin-3 could contribute to metabolic changes that promote tumour growth. The mice develop normally but die prematurely following severe weight loss; this is likely due to reduced caveolae function as mutations in human genes encoding other caveolae-associated proteins are linked to muscle and fat degeneration (Parton and del Pozo, 2013). Precisely how the cellular and metabolic changes observed in cavin-3 depleted cells contribute to the mouse phenotype requires further research.

A major challenge for the future is to understand the cellular roles of distinct caveolae-associated protein complexes. The composition of the complexes can be altered in response to changes in the local environment or upon disease. In addition, some members of the caveolin and cavin families are only expressed in certain types of tissue, indicating that caveolae have cell-type specific functions (Bastiani et al., 2009; Hansen and Nichols, 2010). This is highlighted by recent work showing that, in contrast to the situation in fibroblasts, loss of cavin-3 does not affect the number of caveolae present in endothelial cell membranes (Hansen et al., 2013). It remains unknown whether there are changes in the balance of ERK and Akt signalling or in glucose metabolism in the endothelial cells. Cavin-3 is ubiquitously expressed; however, these findings suggest that it has cell-type specific functions that depend on its interaction with other caveolae proteins. This is supported by observations that cavin-3 is found in protein complexes of varying sizes in different tissues (Hansen et al., 2013).

The study by Michaely and colleagues contributes to the increasingly complex picture being drawn of how caveolae function and provides new insight into their regulation of intracellular signalling. The disruption of caveolae appears to contribute to many diseases including muscular dystrophies, cardiac disease and cancer. Therefore, a detailed understanding of the molecular mechanisms involved may provide potential avenues for therapeutic intervention.
